# *Echinococcus multilocularis* infection affects risk-taking behaviour in *Microtus arvalis*: adaptive manipulation?

**DOI:** 10.1017/S0031182024000507

**Published:** 2024-06

**Authors:** Matilde Martini, Teila Cioli, Thomas Romig, Anna Gagliardo, Dimitri Giunchi, Marco Zaccaroni, Alessandro Massolo

**Affiliations:** 1 Department of Biology, Ethology Unit, University of Pisa, Pisa, Italy; 2 Department of Biological, BIOME Unit, Geological and Environmental Sciences, University of Bologna, Bologna, Italy; 3 Parasitology Unit, Institute of Biology, University of Hohenheim, Stuttgart, Germany; 4 Department of Biology, University of Florence, Florence, Italy; 5 Faculty of Veterinary Medicine, University of Calgary, Calgary, AB, Canada; 6 UMR CNRS 6249 Chrono-environnement, Université Franche-Comté, Besançon, France

**Keywords:** Behavioural alteration, *Echinococcus multilocularis*, *Microtus arvalis*, predator-prey relationship, trophically-transmitted parasites

## Abstract

Manipulation of host behaviour by parasites to enhance transmission to the next host is a fascinating phenomenon that has interested scientists since the 1970s. It has been proposed that infection with the cestode *Echinococcus multilocularis* produces an impairment of the antipredatory behaviour in the rodent intermediate host common vole, *Microtus arvalis*, which may facilitate transmission of the tapeworm to the canid final host. In this study, we observed the behaviour of infected common voles at 12 weeks post-infection, when protoscoleces production and maturation commonly occurs, in order to assess behavioural changes compared to uninfected controls, that might ease predation in the wild. Infected and uninfected voles were monitored for 24 h to observe their spontaneous activity. In addition, the next day, both infected and uninfected voles were subjected to 4 different behavioural tests: open field test, barrier test, platform test and air-puff test in a running wheel. No significant difference between uninfected and infected voles emerged during the behavioural tests. However, observation of spontaneous activity revealed that infected voles increased their feeding frequency and spent significantly more time above bedding even when not eating, compared to the uninfected controls. In the wild, these behavioural changes increase the animals exposure to predators, raising their chance of becoming prey. These findings are the first direct evidence consistent with behavioural manipulation by *E. multilocularis* on common voles.

## Graphical abstract

**Figure:**
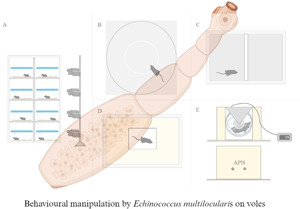

## Introduction

Understanding the transmission dynamics of zoonotic parasites is essential to design efficient public health control strategies. The relationship between a parasite and its host(s) has been the subject of many investigations. For some parasites completion of the life cycle occurs within a single host (simple life-cycle parasites; SLCs), whereas others need different host species (complex life-cycle parasites; CLCs) (Brown *et al*., 2001). For the latter, 1 or more hosts acts as Intermediate Host (IH), in which the parasite reproduces asexually, and one host becomes the Definitive Host (DH), in which the parasite reaches sexual maturity (Brown *et al*., 2001). For a CLC parasite, the most common form of host-to-host transmission is trophic, in which the prey plays the role of IH and the predator DH (Bethel and Holmes, 1973). In the 20th century, the so-called ‘manipulation hypothesis’ was proposed to explain the evolution of the parasite–host relationship in some CLC parasites. This hypothesis predicts that the ability of a parasite to induce phenotypic alteration in the IH produces increased parasite transmission rates to the DH final host (Dobson, 1988; Freedman, 1990; Moore, 2002). The ‘manipulation hypothesis’ is supported by several studies showing an advantage for the parasite presenting an infection-induced phenotypic alteration (e.g. behaviour, morphology and/or physiology) in the IH (Bethel and Holmes, 1973; Lafferty and Morris, 1996; Lefèvre *et al*., 2009).

*Echinococcus multilocularis* (Leuckart, 1863), the third most relevant human food-borne parasite in the world (FAO/WHO, 2014), is a cestode presenting a CLC with IH-DH transmission through predator-prey relationship and could represent a model to study parasite transmission strategies. *E. multilocularis* is a gastrointestinal parasite whose adult develops in the small intestine of wild and domestic canids (e.g. coyote *Canis latrans*, red fox *Vulpes vulpes*, raccoon dog *Nyctereutes procyonoides*, golden jackal *Canis aureus*, wolf *Canis lupus*, domestic dog *Canis lupus familiaris*) and, to a lesser extent, in cats (*Felis catus*) (Romig *et al*., 2017). In the intestinal lumen of the DH, the adult worm produces fertilized eggs which are shed in the environment with the host feces (Romig *et al*., 2017). If accidentally ingested by a competent IH (mostly rodent), the eggs hatch in the host's gastrointestinal tract, releasing an oncosphere which migrates to the target organs, most frequently the liver, through the bloodstream of the portal vein (Eckert *et al*., 2011). Once in the liver, the oncosphere develops into the metacestode (Heath and Lawrence, 1976) which starts asexual multiplication, causing multilocular cyst-like lesions (Kamiya, 2008) and produces the infectious larval stage: the protoscolex. The cycle is completed only when a competent predator consumes an infectious prey (i.e. with fertile lesions containing mature protoscoleces).

Although it has been hypothesized that *E. multilocularis* infected rodents might have a diminished ability to escape predation, so far there are no data supporting such a hypothesis (Vervaeke *et al*., 2006). Therefore, clarifying the effects of *E. multilocularis* infection on IH anti-predatory behaviour may be a further key feature to consider in the epidemiology and transmission studies of this parasite. The overall aim of this study was to preliminarily investigate possible behavioural alterations caused by *E. multilocularis* in the common vole *Microtus arvalis,* a key host species for the maintenance of the parasitic cycle in Europe (see Oksanen *et al*., 2016 and references therein). With this pilot study, we aimed to obtain the proof of concept to support the hypothesis of parasitic manipulation of IH behaviour in *E. multilocularis* so as to facilitate its transmission. Specifically, that if any behavioural alteration is detected, this should be in the direction of an increased risk to predation of infected animals compared to controls; this in the natural cycle would translate into increased transmission rate. Our working hypotheses were: (i) the parasite induces alteration of specific behavioural patterns of the IHs, facilitating its predation (behavioural alteration); (ii) this alteration occurs when the IH is infective (i.e. with viable protoscoleces larvae).

## Materials and methods

### Experimental animals

The experimental groups included 16 *M. arvalis* aged between 9 and 14 weeks, bred and kept in captivity at the Unit of Parasitology of the University of Hohenheim, Stuttgart, Germany. The animals were randomly assigned to 1 of 2 experimental groups: one (treated, T) consisted of 10 animals (5 males and 5 females) infected with 500 viable embryonated eggs of *E. multilocularis*, whereas the other (control, C) was made of 6 sham-treated animals (3 males and 3 females). One infected and 1 control animal died or had to be euthanized by CO_2_, according to legal requirements, before the experiment at 12 weeks post infection (w.p.i.). Therefore, the experiment was done with 14 animals: 9 infected (4 male, 5 female) and 5 control animals (2 male, 3 female). Animals were individually housed, under standard conditions of 12 hours of daylight and 12 hours of darkness (12-12 DL); Light (L): 7 a.m.-7 p.m.; 22°C; humidity 40–60%, in standard polycarbonate cages (Type III: 42.0 × 26.5 × 15.5 cm). Cages were provided with bedding (wood shavings and hay) that was changed once a week. Water and food pellets (pellet food for rats and mice; Altromin®, Germany, Lage) were available *ad libitum*.

### Experimental infection

*E. multilocularis* eggs were obtained from adults collected from the intestines of naturally infected foxes hunted in Switzerland. Before inoculation, the viability of the eggs was tested using the ‘Sodium Hypochlorite Resistance’ test (Joekel and Deplazes, 2017), resulting in a viability of 12.5%. Each treated vole was infected with about 500 eggs by oral gavage, using a metal feeding needle, with the viable eggs suspended in 0.5 ml of PBS. The control animals were sham-inoculated with 0.5 ml of distilled water. After the infection, the voles were housed under BIO III security level for 3 days due to the risk of regurgitation of parasite eggs. After this period, the animals were brought back to the Animal Facility and monitored daily for post-inocula trauma or illness.

### Behavioural observation and tests

All behavioural observation and tests were performed at 12 w.p.i., to allow for protoscolex (infectious larva stage) production and maturation (Woolsey *et al*., 2015). Initially, we planned to carry out behavioural observations also at 18 w.p.i., but due to progressing disease several animals had to be euthanized and the remaining group size did not allow for adequate replication.

**Figure 1.**
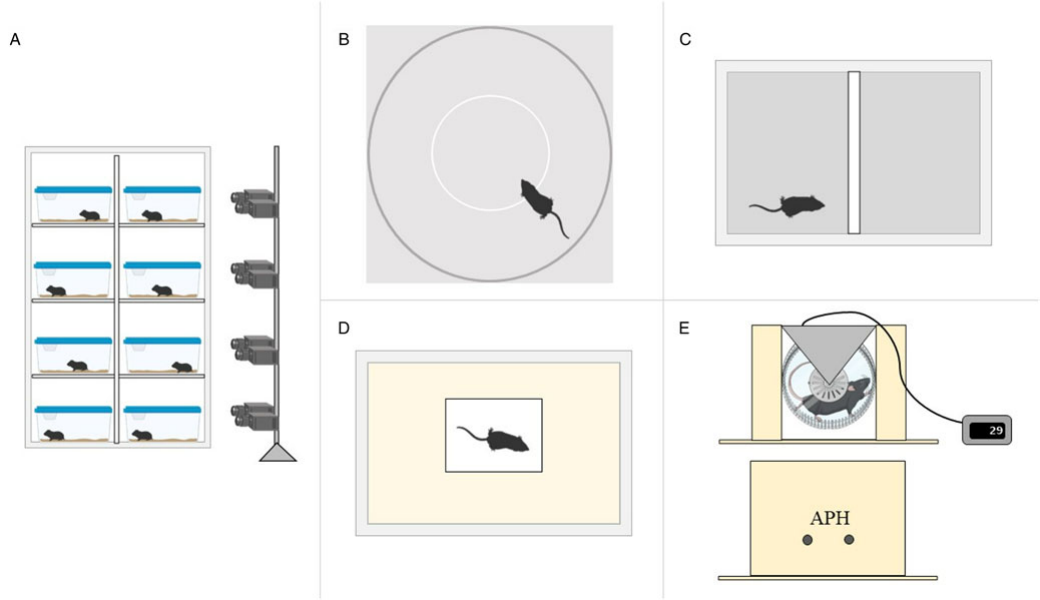
Schematic drawing of the behavioural observation and tests conducted on 9–14 weeks old males and females *Microtus arvalis* to test behavioural manipulation by *Echinococcus multilocularis* infection. Structure of (A) Behavioural observation (24-h monitoring) apparatus; (B) Open field test; (C) Barrier test; (D) Platform test; (E) Running wheel and air puff test (APH, air puff holes). Treated group was subjected to oral injection of 500 *E. multilocularis* eggs (estimated viability of 12.5%), whereas control group was sham-inoculated with distilled water. Figure was created with BioRender.com.

Prior to the experimental phase, the voles were observed for 24 hours to assess spontaneous behaviour in the cage. Afterwards, the animals were subjected to 4 behavioural tests: open field, barrier test, platform test and air puff test in a running wheel (Fig. 1). All tests targeted behaviours potentially associated with anti-predatory strategies. Both open field and barrier tests were conducted to observe exploratory and risk-taking behaviours. The platform test aimed at assessing an anti-predatory response. The observation in a running wheel system aimed at assessing the physical performance, whereas the air puff test, conducted when the animal was stationary in the running wheel, aimed to assess the vole reaction time to a physical stimulus. Many behavioural variables were chosen as recommended by a previous study of Herde and Eccard (2013). All behavioural tests were carried out in succession, in a different room from the one in which the animals had been kept till then, but under the same conditions (12-12 DL, L: 7 a.m.-7 p.m.; 22 C° and 40–60% humidity). The animals were allowed to rest in a cage for 2 h between each trial. All tests were video-recorded, and operators left the test room during the trials.

### Behavioural observation (24-h monitoring)

The spontaneous behaviour of treated and control voles was recorded for 24 h at 12 w.p.i. starting from 6 a.m. To increase the view of the animal in the home cage during the observations, the hay, but not the bedding, was removed from each cage 3 days before the start of monitoring, to accustom the animal to its absence. The 24-h observation was carried out using a night-vision surveillance video camera (Lorex, Flir 8-Channel 1080p DVR) per cage (Fig. 1A). The setup allowed monitoring a maximum of 8 home cages simultaneously, so 2 sessions were needed, 1 day apart. The monitoring was carried out under controlled conditions (12-12 DL, L: 7 a.m. –7 p.m.; 22°C, and 40–60% humidity). No operators entered the room during the 24 h of monitoring. The time spent above the bedding and the frequency and duration of eating and drinking events were recorded (see Table 1).

**Table 1.** List of tested behavioural variables for the in-cage monitoring and behavioural trials performed in the study conducted on 9–14 weeks old males and females *M. arvalis* to test behavioural alteration by *E. multilocularis* infection

Test	Variable	Description
Behavioural observation (24-h monitoring)	Drinking	Events and duration in minutes that the animal spends sucking the bottle of water
	Eating	Events and duration in minutes that the animal spends chewing the pellet
	Above bedding	Total duration in hours that the animal spends above bedding
Open Field test	Peripheral time	Total duration in seconds that the animal spends in the peripheral (safe zone) of the arena
	Centre time	Total duration in seconds that the animal spends in the centre (unsafe zone) of the arena
Barrier test	Freezing	Events and duration in seconds that the animal spends completely stationary in the two sides of the barrier for at least 1 sec
	Latency	Latency (in seconds) to jump over the barrier
	Digging	Events and duration in seconds that the animal spends using its paws to move the sand
Platform test	Latency to hide	Latency (in seconds) to hide into the bedding the first time
Running wheel and air puff test	Spontaneous activity	Presence, events and duration in seconds that the animal spends running into the wheel before the stimulus application
	Freezing after stimulus	Duration in seconds of freezing reaction within 1 min after the stimulus application
	Reaction	Presence of reaction (running) within 3 sec after the stimulus application

Treated group was injected with 500 *E. multilocularis* eggs (estimated viability of 12.5%), whereas control group was sham-inoculated with distilled water. The variables reflect mainly anti-predatory behaviours and activity of the tested animals.

### Open field test

According to Herde and Eccard (2013), the open field test apparatus was built as an arena consisting of a round aluminium box (100 cm diameter) with an open top. The grey wall of the arena was 35 cm high, preventing any animals from escaping. A circle of 80 cm in diameter was drawn around the centre of the arena on the white floor of the arena so as to allow the identification of 2 areas: a safe peripheral zone near the outer wall (a 20 cm wide ring) and an unsafe zone (the centre of the arena; Figure 1B). Areas far from walls are known to be perceived as ‘unsafe’ for small mammals (Prior and Sachser, 1995). Each animal was transported in the test room in a white polycarbonate plastic tube (5.08 × 10.16 cm) and placed in the centre of the arena. The test lasted 10 min and started when the animal reached the arena wall for the first time. The time spent by the vole in each of the 2 areas was recorded (see Table 1).

### Barrier test

The barrier test consisted of a semi-transparent plastic box (36 × 26 × 21 cm) with sterilized sand as bedding. The plastic box was divided into 2 equal compartments by a white, 4.5-cm high wood barrier (Fig. 1C). Each animal was carried to the test room in a white polycarbonate plastic tube (5.08 × 10.16 cm). The animal was randomly placed in 1 of the 2 compartments and the latency to cross the barrier the first time was measured. The duration of the test was 5 min. The other measured variables are reported in Table 1.

### Platform test

The platform test was conducted in a Type IV cage (59 × 380 × 200 cm) with a small white platform (an overturned box of 30 × 17.5 × 6 cm) put in the centre of the home-cage filled with 3 cm of bedding layer (Fig. 1D). Each animal was transported in the test room in a white polycarbonate plastic tube (5.08 × 10.16 cm). The test started when the animal was placed in the centre of the platform and the latency to hide in the bedding was measured. The test ended after 3 min.

### Running wheel and Air puff test

The apparatus used in this test consisted of a voluntary running wheel (11.5 cm diameter). The vole was forced to stay in the running wheel by 3 wooden walls (1 large wall measuring 17 × 14.5 cm, and 2 small walls measuring 11 × 14.5 cm) covered with black cardboard and 1 transparent plastic wall allowing video-monitoring during the test. The air puff was delivered through 2 holes (3 mm diameter) in the largest wooden wall located at 4.5 cm from the bottom of the wheel, so that the air puff stimulus (delivered from compressed air bottle, Air Duster 125 ml, Vivanco®, Germany, Ahrensburg) reached the right side of the vole (Fig. 1E). The number of rotations was recorded by a Liquid Crystal Display (LCD) activity counter (STARR®, life science group; United States, Oakmont) connected to the running wheel. In the described apparatus, the animal was forced to stay inside the wheel, but unlike the standard forced running system, it was free to stay still or move. Each animal was transported in the test room in a white polycarbonate plastic tube (5.08 × 10.16 cm). The test started when the animal was placed in the running wheel. The air puff was delivered when the vole was stationary. The animal received an air-puff stimulus of 1 sec after at least 5 min on the running wheel. The vole was video-monitored from the beginning of the test till 1 min after the stimulus application. The spontaneous activity in the running wheel before the stimulus application and the freezing time after the air puff were recorded. The variables measured are listed in Table 1.

### Post-mortem analyses

All the experimental animals were sacrificed after 18 weeks by CO_2_, according to legal requirements, and subjected to a *post-mortem* visual inspection of the lesions. For estimating the number of mature protoscoleces in each subject the portion of the liver presenting lesions was cut off and homogenized using a pestle in 1 ml of distilled water. The homogenate was filtered through a nylon mesh of 1 mm pore size (the filter was washed with 1 ml of distilled water) onto a squared petri dish (12 × 12 cm) with a 5 × 5 mm squared grid on the bottom for a total of 576 cells. The protoscoleces inside the cells of the grid laying on both diagonals of the petri dish (48 cells) were manually counted under a microscope. An estimate of the number of protoscoleces was then computed by multiplying the average number of protoscoleces calculated for the inspected cells by the total number of cells.

### Behaviour quantitative analysis

The videos were exported in .*asf* format and imported using Boris version 6.3.5 software (Friard and Gamba, 2016). Each video was checked at normal speed, and the total duration in seconds (state behaviour) and the occurrence of an event(s) of each variable (event behaviour) was considered. Software R 4.2.2 (R Core Team, 2022) was used for all data analyses. Each variable reported in Table 1 was compared between infected and control groups using a 2 sample bootstrap *t*-test (Efron and Tibshirani, 1994) by means of the *boot.t.test* function in the package *MKinfer* (Kohl, 2019), setting the number of bootstrap samples = 10 000. This resampling approach was chosen as it is more robust and powerful with small and unbalanced samples, as well as for hypothesis testing for groups with different patterns of non-normality but equal variance (Dwivedi *et al*., 2017). Sex was excluded from the analysis because we did not expect a differential effect of infection on different sexes (Woolsey *et al*., 2015). Furthermore, a Fisher exact test was performed to compare the frequency of infected and uninfected animals that showed spontaneous activity in the wheel, before the application of the air puff stimulus. The same test was also applied to compare the frequency of infected and uninfected vole behavioural reaction within 3 sec after the air puff stimulus application. We used an alpha level of 0.05 for all statistical tests.

## Results

### Behavioural observation (24-h monitoring)

**Figure 2.**
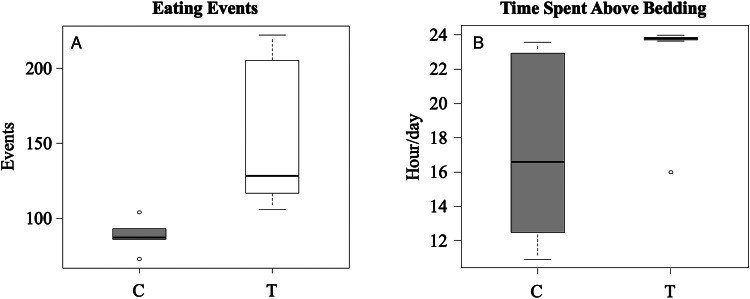
Results of 24-h behavioural observation conducted on 9–14 weeks old males and females *Microtus arvalis* to test behavioural manipulation by *Echinococcus multilocularis* infection. Boxplots of: (A) Eating events; (B) Time spent above bedding within 24 h (duration). Treated group (white colour) was injected with 500 *E. multilocularis* eggs (estimated viability of 12.5%), whereas control group (grey colour) was treated only with distilled water.

During the 24-h in-cage monitoring period, infected voles spent, on average, 5.6 h more above bedding compared to intact voles (32.4% increase, T-voles = 22.93 ± 0.87 *vs* C-voles = 17.29 ± 2.61, *P* = 0.03; Table 2, Fig. 2B). Furthermore, infected voles showed a mean increase of 62.5 eating events per 24 h compared to control animals (41.37% increase, T-voles = 151.11 ± 15.52 *vs* C-voles = 88.60 ± 5.05; *P* = 0.002; Table 2, Fig. 2A).

**Table 2.** Results of the bootstrap *t*-test calculated on the variables measured in the behavioural tests on 9–14 weeks old males and females *M. arvalis* aimed at testing behavioural manipulation by *E. multilocularis* infection

Test	Variables (measure unit)	Difference of means	95% CIs		*P* value
			Lower	Upper	
24-h monitoring	Drinking Events (frequency)	−2.6	−38.2	34.2	0.9
	Drinking Duration (minute)	−8.5	−20.9	3.9	0.2
	Eating Events (frequency)	−62.3	−110.9	−8.7	**0**.**002**
	Eating Duration (minute)	1.8	−17.4	20.4	0.9
	Above Bedding (hour)	−5.6	−10.8	−0.9	**0**.**03**
Open field test	Peripheral time (second)	2.1	−16.3	19.4	0.8
	Centre time (second)	−2.3	−19.3	16.4	0.8
Barrier test	Freezing Events (frequency)	3.5	−1.0	8.7	0.2
	Freezing duration (second)	6.5	−48.5	61.6	0.8
	Latency (second)	−41.5	−127.0	40.9	0.3
	Digging events (frequency)	−0.7	−6.5	5.9	0.9
	Digging duration (second)	−7.0	−42.9	34.3	0.8
Platform test	Latency to hide (second)	−10.9	−44.5	29.5	0.7
Running wheel and air puff test	Spontaneous activity events (frequency)	0.04	−5.5	5.7	0.9
	Spontaneous activity duration (second)	−25.1	−88.1	39.9	0.5
	Freezing after stimulus (second)	−8.2	−37.5	21.0	0.6

Treated group was subjected to oral injection of 500 *E. multilocularis* eggs (estimated viability of 12.5%), whereas control group was sham-inoculated with distilled water. The bootstrap *P* value is based on 10 000 bootstrap replications. Significant *P* values (*P* < 0.05) are in bold. Difference of means = control group mean – treated group mean.

### Behavioural tests

No significant differences between control and treated voles were found for any of the behavioural tests (open field, platform, barrier, running wheel) (Table 2). Moreover, in spontaneous running activity in the wheel, infected and uninfected voles did not show any significant difference (Fisher exact test, *P* > 0.05). In fact, all control voles displayed spontaneous running activity in the running wheel before the air puff test, as did 8 out of 9 infected voles. As regards the voles that engaged in some spontaneous running, we did not detect any significant difference between control and treated voles in the time they spent doing this activity and the running events (Table 2). The most common reaction to the air puff observed within 3 sec from the stimulus was a freezing reaction (3 control and 7 infected voles froze after the stimulus; Fisher exact test, *P* > 0.05). In addition, the time spent freezing in the 60 seconds after stimulus delivery did not differ in the 2 experimental groups (Table 2).

### Post-mortem analyses

Seven of the infected voles were euthanized and necropsied at 18 w.p.i., 2 had to be euthanized and necropsied earlier due to disease progress in line with animals welfare requirements (13 w.p.i.). All infected voles had *E. multilocularis* lesions in the liver and mature protoscoleces. One animal necropsied at 13 w.p.i. presented 5 discrete lesions with 44 904 protoscoleces, 1 necropsied at 13 w.p.i. had 3 discrete lesions with 10 836 protoscoleces, while animals necropsied at 18 w.p.i. presented an average ± s.e. of 2.7 ± 0.9 lesions each with a mean ± s.e. of 113 773 ± 59 431 (min = 1342, max = 452 687) mature protoscoleces, *N* = 7. Lesions were present in all the liver lobes, with 9 lesions found in the Left Lateral Lobe, 4 in the Left Medial Lobe, 6 in the Right Medial, 7 in the Right Lateral Lobes and 1 in the Caudate Lobe. All control voles were euthanized 15–18 w.p.i., no lesions and protoscoleces were present in any of these animals.

## Discussion

Our study aimed to assess whether and which behavioural alterations might occur following *E. multilocularis* infections in one of its IHs, *M. arvalis*. Specifically, we compared the behaviour of voles experimentally infected with *E. multilocularis* eggs to the behaviour of uninfected ones at 12 weeks post-infection.

Both infected and uninfected voles displayed comparable behaviours when tested in arenas, experimental cages, or running wheels. However, despite the small sample size, a clear difference emerged between the 2 groups in spontaneous behaviours in their home cage. In fact, we observed a significant increase in the time spent above bedding by infected animals compared to intact voles (Fig. 2B). This noteworthy result highlights that the condition of being infected may have affected the animals’ hiding behaviour, which is their spontaneous anti-predator response (Sundell and Ylönen, 2004), resulting in a significant increase in time spent in the open space of the cage. Moreover, the infected voles, when above bedding, fed more frequently compared to the controls (Fig. 2A). In the wild this behavioural alteration would translate to longer time spent in the open, outside the nest, and more frequent feeding with an increased likelihood of predation by a DH. This is consistent with the hypothesis that the parasite can induce behavioural modification, making the IH more susceptible to predation. This conclusion is in line with previous studies reporting that cestodes alter IH's activity by decreasing or increasing the host activity level depending on the species involved (Lafferty and Shaw, 2013). For instance, *Echinococcus granulosus* (*sensu lato*) infection may cause a moose physical impairment due to lung lesions induced by the parasite larval stage (Messier *et al*., 1989). This case can be explained by the side effect of being infected (Dantzer *et al*., 2008; Adamo, 2013) rather than a manipulation of the parasite on the IH. Differently, fishes infected by *Schistocephalus solidus* larval stages display increased surfacing activity, consequently increasing the probability of being detected by a predator (Øverli *et al*., 2001). In this case the effect of the parasite can be more plausibly explained by a specific manipulation aimed at increasing the transmission probability. This seems to be the same effect we observed in our voles infected by *E. multilocularis.* As a matter of fact, spontaneous activity in the running wheel (before the air puff test) did not differ between infected and uninfected voles, suggesting that the parasite did not seem to cause (at least up to 12 weeks from infection onset) a general debilitation of the voles. Furthermore, the freezing reaction, an anti-predatory strategy displayed by rodents, is triggered by traumatic or stressful experiences (Roelofs, 2017). Both control and treated animals comparably showed freezing reaction to the air puff stimulus, suggesting that the parasite infection did not significantly alter fear elicited behavioural responses.

Although we did not measure the amount of food eaten by intact and infected animals when above bedding, it would not be surprising if the more frequent feeding of the infected voles resulted in greater food consumption. However, the observed increase in frequency of feeding might be consistent with 1 or 2 of two different mechanisms linked to an increase in energy demand in infected animals: the parasite's direct draw of energy from the host or the host's immune response to the infection (Schmid-Hempel, 2005). Referring to the former mechanism, an alteration of the insulin pathway and, possibly, of the glucose levels might induce a nutritional needs impairment and an alteration of food intake requirements. It is known that *E. multilocularis* has receptors for insulin to target the liver as an optimal environment for the settlement and establishment of metacestodes (Hemer *et al*., 2014). However, it is unknown whether the parasite might have nutritional dependence on host glucose, as is the case of the trematode *Schistosoma mansoni* (Ahier *et al*., 2008) or what the role of insulin receptors in modulating the glucose parasite level could be (Brehm *et al*., 2006). In addition, the time spent above bedding might be altered *via* physiological systems other than the digestive system. For instance, the parasite might increase the host oxygenation demand, as studied in swine affected by pulmonary metastrongylosis, a parasitosis caused by nematode *Metastrongylus* species (Pavlović *et al*., 2021). Another example of pulmonary debilitation which involves the helminth-amphibian host system, regards infected toads (*Bufo bufo*) which might have decreased lung function and, consequently, an altered oxygenation demand (Goater and Ward, 1992). In our case, the altered oxygen demand can directly induce the vole to stay above bedding almost all the time. In this case, the increased feeding frequency might be caused simply by the continuous availability of food close to the animal. Another possibility could be that liver tissue damage (in this case induced by larvae proliferation) might cause a host blood intoxication (Lockwood *et al*., 1979). Toxicants in the blood, such as ammonia, might have a neurotoxic effect, impairing the activity of the IH, its reactivity to stimuli and mobility, thus making it more susceptible to predation (Ong *et al*., 2003; Lockwood, 2004). Our findings are not consistent with this hypothesis as the infected voles do not seem to be less mobile nor less reactive than control voles.

Despite the small sample size and the unclear physiological mechanisms underlying the increased above-bedding activity in *E. multilocularis-*infected IHs, the pilot study provided the first direct evidence of IH behavioural alteration following *E. multilocularis* infection. In particular, the parasite infection increased both the time spent above bedding as well as the time the voles spent feeding compared to the intact controls, suggesting a specific effect of the parasite on the activity of the IH. The study is intended as a starting point for investigating the effect of *E. multilocularis* on the physiology of rodent IHs.

## Data Availability

All data and code used for the analyses are available at GitHub repository link: https://github.com/MatildeUNIBO/ParManPilot.git.
